# Segregation *versus* Interdigitation in Highly Dynamic Polymer/Surfactant Layers

**DOI:** 10.3390/polym11010109

**Published:** 2019-01-10

**Authors:** Omar T. Mansour, Beatrice Cattoz, Manon Beaube, Richard K. Heenan, Ralf Schweins, Jamie Hurcom, Peter C. Griffiths

**Affiliations:** 1 Faculty of Engineering and Science, University of Greenwich, Medway Campus, Central Avenue, Chatham Maritime, Kent ME4 4TB, UK; O.T.Mansour@greenwich.ac.uk (O.T.M.); Beatrice.cattoz@gmail.com (B.C.); pcg1967@gmail.com (M.B.); 2 Science and Technology Facilities Council, ISIS Facility, Rutherford Appleton Laboratory, Didcot, Oxfordshire OX11 0QX, UK; Richard.heenan@stfc.ac.uk; 3 Institut Laue Langevin ILL, 6 rue Jules Horowitz, 38000 Grenoble, France; schweins@ill.eu; 4 School of Chemistry, Cardiff University, Main Building, Park Place, Cardiff CF10 3TB, UK; HurcomJ@cardiff.ac.uk

**Keywords:** Pluronic, surfactants, foams, SANS, multilayers

## Abstract

Many polymer/surfactant formulations involve a trapped kinetic state that provides some beneficial character to the formulation. However, the vast majority of studies on formulations focus on equilibrium states. Here, nanoscale structures present at dynamic interfaces in the form of air-in-water foams are explored, stabilised by mixtures of commonly used non-ionic, surface active block copolymers (Pluronic^®^) and small molecule ionic surfactants (sodium dodecylsulfate, SDS, and dodecyltrimethylammonium bromide, C_12_TAB). Transient foams formed from binary mixtures of these surfactants shows considerable changes in stability which correlate with the strength of the solution interaction which delineate the interfacial structures. Weak solution interactions reflective of distinct coexisting micellar structures in solution lead to segregated layers at the foam interface, whereas strong solution interactions lead to mixed structures both in bulk solution, forming interdigitated layers at the interface.

## 1. Introduction

Polymer-surfactant stabilised foams are of growing interest in a wide range of industries-paper, foodstuffs, home, personal care and pharmaceutical-either because the foam is an end-product or encountered during the manufacturing process. The ability to control the interactions between the polymers and the surfactants provides new approaches to control the foaming properties of these systems, and eventually, optimizing the performance of the formulation. Foams are thermodynamically unstable, and therefore surface active species like proteins, particles, polymers, surfactants and their mixtures are commonly used to stabilise the foam by slowing the drainage, coalescence and coarsening of the foam structures [1]. How this stability is achieved is still not fully understood.

Mixtures of polymers and surfactants are ubiquitous and their bulk and equilibrium interfacial behaviours have been investigated at length. Generally, the systems may be differentiated by the strength of the interactions between the surfactant and the polymer chains, these being hydrophobic and/or electrostatic in nature, depending on the chemical composition of the system. The key surfactant structure in these complexes could be of monomeric or micellar nature depending on several factors, but notably the surfactant/polymer concentration, the presence of any additives and the conditions of the solution being studied [2]. Multi-layer structures are often observed, especially in the context of oppositely charged polymers and surfactants, but less so with non-ionic surface active polymers and surfactants [3,4,5,6,7,8,9,10,11].

Few studies have focused on the relationship between adsorbed layers and the foam stability (time taken for the foam to collapse) and/or “foaminess” (measured height of a column of foam generated under controlled conditions) from a detailed structural analysis of the interfacial layers. One notable study is that of Petkova et al., who investigated foams stabilised by blends of *non-surface active* polymers (poly(vinylamine), poly(*N*-vinyl-formamide)) and small molecule surfactants (SMS) (sodium dodecylsulfate (SDS), C_12_TAB (dodecyltrimethylammonium bromide) and Brij 35 (C_12_EO_23_)) that show strong and weak solution interactions. In these studies, less foamabililty, but higher foam stability was recorded from polymer-surfactant mixtures showing strong synergistic interactions [12].

Recent neutron reflectivity (NR) studies have concluded that the “equilibrium” interfacial structures of surface active species such as surfactants and polymers are rather more complex than historically modelled, and often, experimental findings are difficult to deconvolute. The origin of this is thought to be the formation of multilayer structures [13,14,15], though not all experiments provided unequivocal evidence for this (such as Bragg peaks). However, some studies especially on oppositely charged polymer/surfactant complexes do exhibit these features e.g., Campbell et al. [3,16] and others [8,17,18,19,20]. Further, for oppositely charged systems, characteristics that impact the kinetics of interaction such as the order of mixing, are shown to be dominating factors in defining the structures that ultimately form [10]. Therefore, it is hypothesized that there is an as-yet, an ill-defined relationship between the surface and bulk structures in these slowly equilibrating systems.

We have previously deployed small-angle neutron scattering (SANS) to study foams stabilised by single component solutions of non-ionic polymers of the Pluronic family [21] and small molecule surfactants [22], since neutron techniques have a proven ability to probe the adsorption of molecules at interfaces. Such experimental approaches have contributed significantly to the understanding of the structure activity relationships of interfacial bound species. Of key interest in that work were observations of (Bragg) peaks in the scattering data suggesting the presence of polymer and/or surfactant multilayer structures [23,24,25] at the air-water interface present in wet foams. It is therefore hypothesized that the multilayer structure is induced by the non-equilibrium nature of the foam, and as such these observations resonate with “equilibrium” reflectivity studies on the more slowly equilibrating oppositely charged polymer/surfactant systems. Our data were successfully described by a small number (*M*) of discrete layers of thickness (*L*) and spacing (*D*) [26,27] though it must be said, that multilayer structures at dynamic interfaces is not a universally accepted view [28,29,30,31,32] and further research is warranted. 

Herein, we extend our previous SANS studies to include investigation of the interfacial structure of foams stabilised by two surface active species (and thus, in contrast to Petkova [12]); non-ionic triblock copolymers Pluronic and SMS. To the best of our knowledge, this is the first time that foams stabilised by mixtures of surface active polymers and surfactants has been investigated by SANS, and should complement the reflectively studies on oppositely charged polymer/surfactant systems. Significant changes in the foam stability measurements were also observed as the strength of interactions varied from weak to strong, evident by more stable foams for the systems showing strong or “synergistic” solution interactions (Supplementary Information).

Wet (continuously generated) foams consisting of bubbles ranging in size from a few to tens of millimetres in diameter, with film thicknesses of microns, were prepared using the following SMS; anionic SDS, cationic C_12_TAB, and non-ionic polymeric surfactants Pluronic P123 (EO_20_PO_70_EO_20_) and L62 (EO_6_PO_34_EO_6_). Further, mixtures of Pluronic P123 and SMS at concentrations significantly below the respective critical micelle concentration (CMC) or mixed CMC as measured by surface tensiometry (Figures S5 and S6) to avoid the presence of any solution-like micelles have also been explored.

The SANS data will be presented in the following manner; (a) foams stabilised by a weakly interacting system; Pluronic P123 and C_12_TAB system [33]; (b) foams stabilised by a strongly interacting system; Pluronic P123 and SDS and finally; (c) to show temperature induced micellisation, foams stabilised by Pluronic L62 as a function of temperature. It is hoped that this work would highlight how the structural variations of the commonly used temperature sensitive surface active polymers (Pluronic) and the interactions between these polymers and SMS in bulk affect the surfactant structures at the foam air-water interface.

## 2. Materials and Methods

### 2.1. Materials

Sodium dodecylsulfate (SDS, ≥99.0%) and dodecyltrimethylammonium bromide (C_12_TAB, ≥99.0%), Pluronic^®^ P123 and L62 were purchased from Sigma Aldrich and were used without further purification as the surface tension data did not point to the presence of any impurities (Figures S3 & S4, Table S1). Deuterated sodium dodecylsulfate (d-SDS), dodecyltrimethylammonium bromide (d-C_12_TAB), were synthesised by ISIS deuteration facility and have been used as received. All the samples were prepared in deuterium oxide (D_2_O, ≥99.8%, Sigma Aldrich, Gillingham, UK).

### 2.2. Methods

#### 2.2.1. Tensiometry

Surface tension measurements were carried out using a maximum bubble pressure tensiometer (SITA science on-line t60, Germany), calibrated by reference to de-ionized water. Surface tension was recorded at a bubble lifetimes of 10 s. All the CMC determination measurements were taken at 25 ± 1 °C, Table S1. The CMT determination measurements for Pluronic L62 were performed at concentration of 2 wt% and a temperature range of 20 °C to 50 °C, Figure S7.

#### 2.2.2. Foam Stability Measurements

Measurements were carried out in a graduated glass column, 45 cm in height and 20 mm in diameter, Figure S1. The column has a porous frit disk (porosity of 2 µm) placed at the bottom of the column. The airflow was controlled via a flow meter. 2.5 cm^3^ of the surfactant solution was placed at the bottom of the column and nitrogen gas was passed through the sample (constant flow rate of 0.04 L/min and 0.4 bar pressure). Foam with a standard height of 15 cm was generated, after which the gas flow was turned off and the foam was allowed to drain and collapse. The time taken by the foam to drop to half of its original height is defined as the half-life. All measurements were recorded twice; new aliquots of surfactant solution were used for each foam test and the column was washed with deionised water and dried between each test to ensure reproducibility.

#### 2.2.3. Small-Angle Neutron Scattering (SANS)

SANS experiments were performed on either (i) the time of flight *SANS2d* diffractometer at the ISIS pulsed spallation neutron source, Rutherford Appleton Laboratory, Didcot, UK. A range defined by *Q* = (4*π*/*λ*) sin (*θ*/2) between 0.005 and ≥ 0.3 Å^−1^ was obtained by using neutron wavelengths (*λ*) 1.75 to 16.5 Å with a fixed detector distance of 4 m or (ii) steady-state reactor source, *D11* diffractometer at the *ILL*, Grenoble where a *Q* range is selected by choosing three instrument settings at a constant neutron wavelength (*λ*) of 8 Å (*ILL*) with a sample detector distance of 1.2, 8 and 39 m.

Experimental measurement times were around 5 min (*SANS2d*) and between 10–15 min (*ILL*, longer as three detector distances were used with no offsets or discontinuities between the various configurations). All scattering data presented in this paper were (a) normalized for the sample transmission; (b) background corrected using the empty foam cell and; (c) corrected for the linearity and efficiency of the detector response using the instrument specific software package and the scattering from a polystyrene blend taped to the front of the foam cell, Figure S2.

##### SANS Data Modelling

**Scheme 1:**
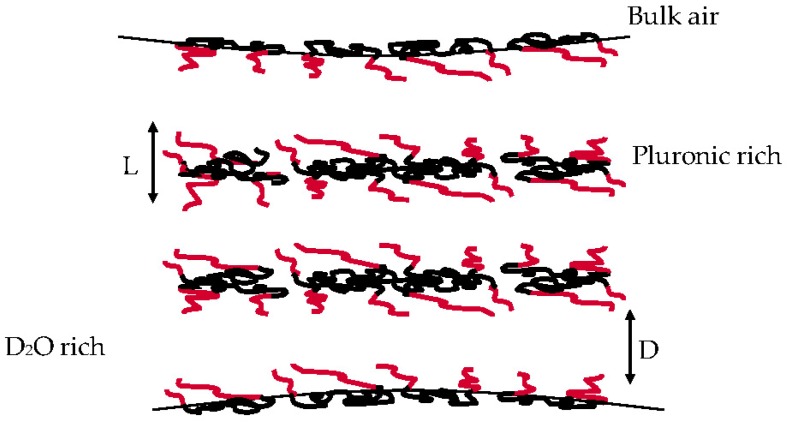
Schematic presentation of the multilayer model of the adsorbed Pluronic layers at the air/water interface. L is the layer thickness and D defines the separation. Not to scale.

Visually, the bubbles studied here are highly curved, comprising spherical pockets of air that are a few to tens of millimetres in size, with the fluid lamellae being easily observable to the naked eye. However, to the neutrons these interfaces are large and flat. The nano-scale structures assembled at these air-water interfaces may be characterized by a model, represented in Scheme 1, comprising *M* thin paracrystalline polymer/surfactant/water multilayers of thickness *L* and separation *D* with diffuseness *T_i_* (the variation in interface structure perpendicular to the interface; an ideal interface will have zero diffuseness). A *Q^n^* term was also added to this model to account for the scattering arising from the smooth air/water interface. To limit the functionality of the fit, the diffuseness *T_i_* has been constrained to 0.01 as per previous studies [26,27]. 

Therefore, in this model, *I*(*Q*) = *I*(*Q*)_lamellar_*S(*Q*), where *I*(*Q*)*_lamellar_* is expressed as:

$$I{\left(Q\right)}_{lamellar}\to N\;{\left(\rho_{1}-\rho_{3}\right)}^{2}V^{2}{\left(\frac{\sin\left(\frac{QL_{1}}{2}\right)}{\frac{QL_{1}}{2}}\right)}^{2}$$

where *N* is the number of scatterers per unit volume, cm^−1^, *ρ*_1_ is the scattering length density (SLD) of the polymer/surfactant layer, *ρ*_3_ is the SLD of the solvent, *V* is the volume of the scatterer and *L* is the thickness of the polymer and/or surfactant layer. The *S*(*Q*) used here is that of a one dimensional paracrystal, Equations (9)–(12) in the model description detailed by Kotlarchyk et al. [26] The main contributing terms to the *S*(*Q*) are *M* and *D* as explained earlier, in addition to a Gaussian distribution term, *σD*/*D*; these give rise to the peaks observed in the reduced (*Q^n^* subtracted) visualization.

Typical starting values for heterogeneity for *L* and *D* expressed as σ(*L*)/*L* and σ(*D*)/*D* are 0.2, though these values have been shown to have a negligible effect on the overall quality of the fit (within reasonable bounds). The SLDs (contrast) of the various materials is such that in D_2_O, the scattering arises equally from the air–D_2_O and polymer/surfactant–D_2_O interfaces, and any further deconvolution of the data is not feasible, at least in these systems. 

The thickness *L* value was estimated by calculating the critical chain length (Å) for the small molecule surfactants (1.5 + (1.26 × *N_c_*) × 2)., where *N_c_* is the number of carbon atoms in the alkyl chain. As for the Pluronic, the thickness *L* was determined from previous neutron reflectivity studies but also taking into consideration the sensitivity of the model’s parameters. The value for the number of layers (*M*) necessary to produce suitable fits was around *n* ≈ 5), larger values did not significantly improve the quality of the fit. No significant difference was observed between the *d-spacing* values obtained from the visual inspection of the scattering data and the values obtained for *D* from the fitting routine.

## 3. Results and Discussion

**Figure 1:**
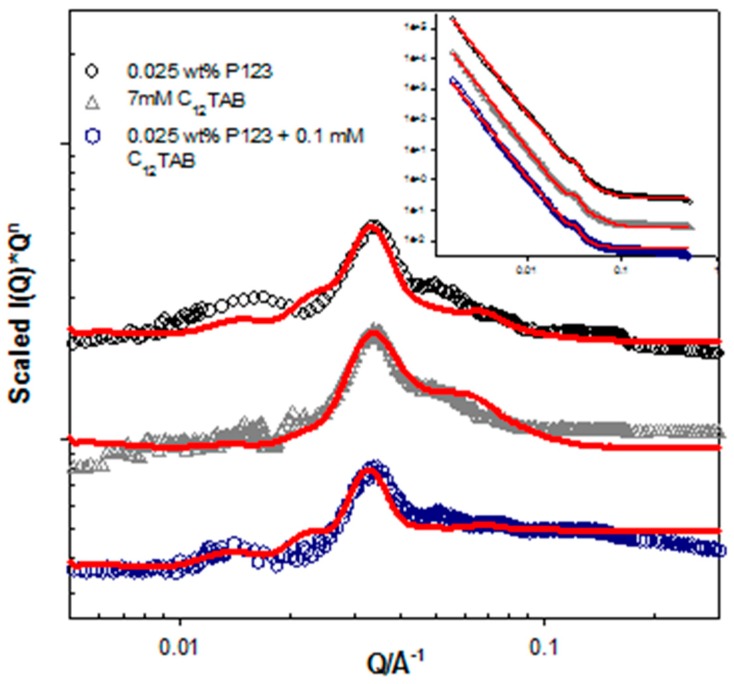
Small-angle scattering recast into an *I*(*Q*)*Q^n^ vs. *Q* format arising from foam stabilised by Pluronic P123, C_12_TAB and their mixture, recorded on the *D11* diffractometer. Data have been offset for clarity. The inset figure presents the raw data. Solid lines are fits to the multilayer model. Both the main figure and the inset are presented in a double logarithmic representation. All samples were prepared in D_2_O.

The measured SANS data from these systems, the insets in Figure 1 (and also in Figure 3), show a pronounced *Q*^−4^ dependence, as expected from the intense Porod surface scattering from the large smooth surface of the bubbles. A visualisation strategy in which the data is plotted in a *I*(*Q*)*Q^−*n*^ representation was developed, (*n* = 4 ± 0.05), Figure 1 and Figure 3, to reveal the several subtle inflexion(s) in these data observed across the *Q* range. The *Q* position of these inflexions was found to be sensitive to the surfactant and/or polymer structure, and to the level of the interactions between the components. The data were fitted to a multilayer model, as shown in the materials and methods section and the fit is also presented in an *I*(*Q*)_fit_*Q^−*n*^ representation.

SANS from pure P123 at 0.025 wt% (half of the measured CMC), Figure 1, show that these inflexions corresponds to *d-spacings* (2π/*Q*) of ≈ 370 Å (*Q* ≈ 0.017 Å^−1^) and ≈ 180 Å (*Q* ≈ 0.0347 Å^−1^), Table S2. These *d-spacing* values were consistent in both data sets acquired from the two different diffractometers used in this study (*SANS2d* and *D11*).

The peak at mid *Q* (≈ 0.03 Å^−1^) is usually the most discernible. The presence of what could be interpreted as a higher order peak at the lower *Q* value can be related to the regular arrangement of the surfactant multilayers, albeit within fractured or heterogeneous lamellar structures. For a perfectly lamellar structure, one would expect to see regular reflections (*n* = 1, *n* = 2, *n* = 3) however, the subtle differences observed in the peak positions implies that the structure is not perfectly lamellar (in the direction normal to the interface).

Both P123 datasets (*SANS2d* and *ILL*) have been successfully fitted to the multilayer model, Table 1. The fit revealed a Pluronic layer thickness of 140 Å and a *D* of 180 Å, however for the *SANS2d* data set, the fit was able to capture both low and mid *Q* peaks with a *D* value of 390 Å, in good agreement with our previous work [21]. Clearly, the model captures the gross features in the data very well, namely the peak position (especially that of the main peak), but it also captures subtleties in the data, such as the weaker shoulders evident in the Porod plots.

Upon introducing C_12_TAB to the system, no significant change in the peak position at mid *Q* is observed. In the foam stability studies, the stability of the foam formed from these systems is (only) slightly reduced (supplemental information). The non-changing position of this inflexion from the foam stabilised by the mixture of P123 (0.025 wt%) and C_12_TAB (0.1 mM), when compared with foams from both the pure systems of P123 (0.0347Å^−1^) and C_12_TAB (0.0370 Å^−1^), indicates the conclusion proposed by Petkova et al. [13] regarding the correlation between the weak interactions observed in bulk and dynamic interfacial structures is more general i.e., it pertains to other non-surface active polymer/surfactant mixtures as well as surface active polymer/surfactant mixtures. The data fitting results are also in agreement with these conclusions, Table 1.

One can postulate that the thinner C_12_TAB (40 Å; 1.5 + (1.26 × *N_c_*) × 2) layers are “coexisting” between the segregated thicker P123 layers (140 Å), without a significant change in the dimension and/or the separation of the P123 layers. Such structures would seem logical assuming distinct populations of the two species in bulk solution [33,34] associated with weak interactions between the two components.

This hypothesis was further explored by a contrast variation SANS approach with deuterated C_12_TAB, where the foam scattering is dominated by the P123 (supplemental information). This experiment, Figure S10, shows that for the P123 + h-C_12_TAB system, two peaks are observed at *d-spacing* ≈ 380 Å and 195 Å respectively, but for the P123 + d-C_12_TAB, there is a shift in both peak positions towards lower *Q* values (*d-spacing* ≈ 400 Å and 200 Å). The observation of these larger spacings-with dimensions much more akin to the pure P123 foam—is consistent with the fact that the C_12_TAB is rendered invisible.

Moving to the strongly interacting system, P123 (EO_20_PO_70_EO_20_) and SDS, several published works have shown the formation of mixed micelles at concentrations above the mixed CMC. These micelles were found to be smaller in size (≈28 Å) when compared with pure P123 micelles (≈70 Å). [34] Further, the interfacial structure of the triblock copolymer EO_23_PO_52_EO_23_ in solution was also studied by neutron reflection at different concentrations, where a total layer thickness of 72 Å was noted. Upon addition of SDS, the layer thickness was found to be between 46 and 49 Å [13,14].

**Figure 2:**
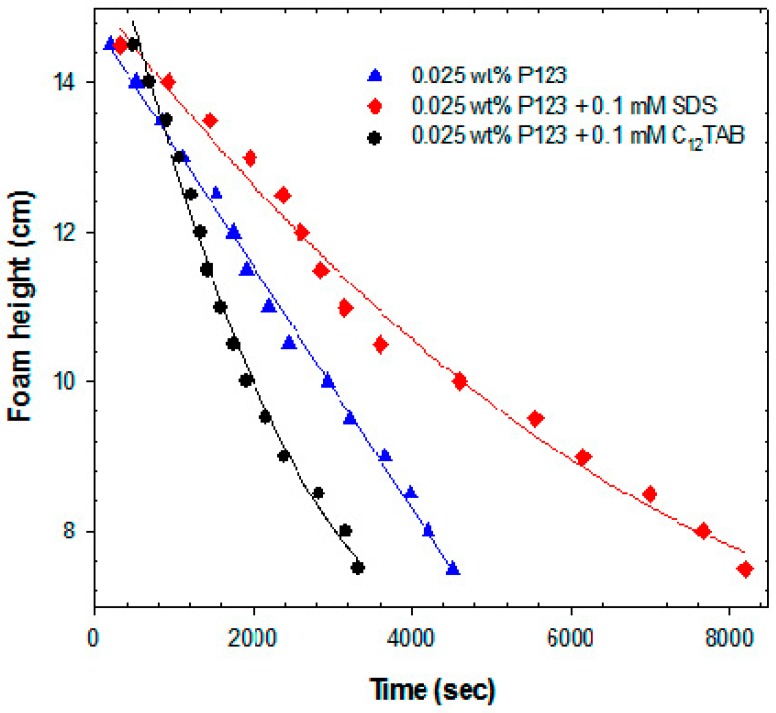
Foam height of Pluronic P123 and SMS mixtures as a function of time.

**Figure 3:**
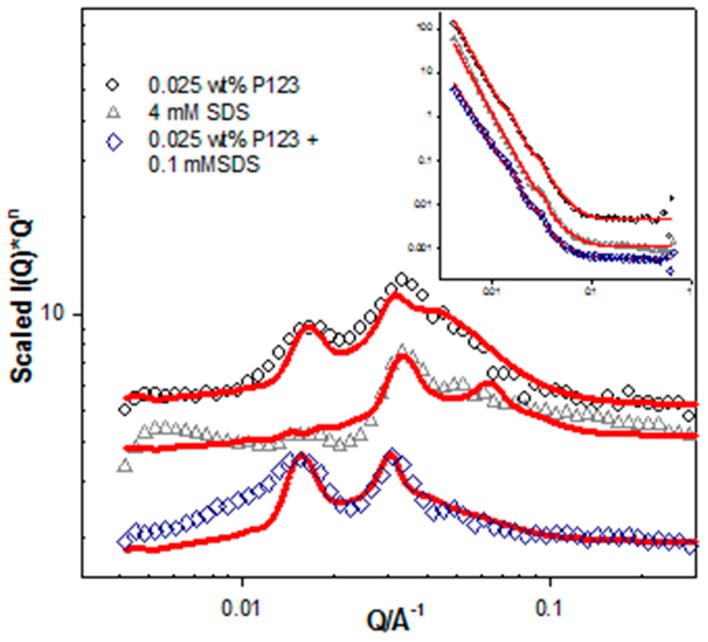
Small-angle scattering recast into an *I*(*Q*)*Q^n^ vs. *Q* format arising from foam stabilised by Pluronic P123, SDS and their mixture, recorded on the *SANS2d* diffractometer. Data have been offset for clarity. The inset figure presents the raw data. Solid lines are fits to the multilayer model. Both the main figure and the inset are presented in a double logarithmic representation. All samples were prepared in D_2_O.

Foams stabilised by the mixture of P123 and SDS showed the most significant change in foam stability, Figure 2, and in the SANS data, Figure 3. For P123 at concentrations equal to half of its measured CMC (0.025 wt%), the decay in the stability profile is rapid with a half-life of ≈4250 s. Upon the addition of a small concentration of SDS, 0.1 mM, the strong synergy between both components leads to a significantly enhanced foam stability, with a half-life that is now almost double that of the P123 only (≈8000 s).

Recall the SANS data from foams stabilised by 4 mM SDS (only) showed one peak at mid *Q* consistent with a surfactant layer thickness of 35 Å. On addition of SDS to the P123 solution, even at concentrations as low as 0.1 mM, there is a significant impact on the position of the P123 peaks. For example, the peak at low *Q,* now corresponds to a *d-spacing* value of ≈ 380 Å (from 370 Å), whereas the peak at mid *Q* corresponds to a *d-spacing* value of ≈ 205 Å (from 180 Å). This was also evident in the data modelling where the layer thickness was found to be 120 Å each (M ≈ 5) with a *D* value of 410 Å.

The increase in both values of the spacing for the P123/SDS case suggests the formation of a new polymer-surfactant structure at the air-water interface, in which the SDS is absorbed or laterally interdigitated within the P123 layers, forming thinner, mixed surfactant layers, resulting in a larger spacing between these layers. This is consistent with the smaller micelles seen in solution, albeit at higher concentrations.

The same approach for data presentation has been followed as before and Porod plots have been used to highlight the peaks at low and mid *Q*, Figure 3. The observations from the SANS contrast variation (d-SDS and P123) measurements, Figure S9, from these systems also reiterate the hypothesis that the peak positions of the d-SDS/P123 system show no significant change from the h-SDS/P123 system, however, both peak positions differ from the pure P123 system.

To further illustrate that these Bragg features arise from interfacial species, foams stabilised by a different molecular weight Pluronic, L62, were also investigated by SANS as a function of temperature (Pluronic P123 has a low critical micelle temperature, CMT; 16 °C), since on passing through the CMT the aqueous phase comprising the interstitial volumes will now contain micelles. 

**Figure 4:**
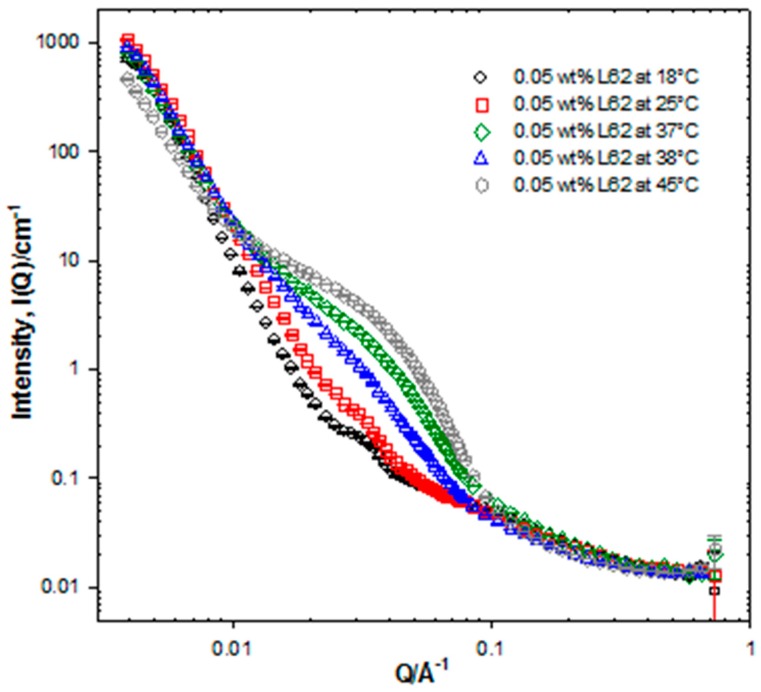
Small-angle scattering presented in the conventional, double logarithmic *I*(*Q*) vs. *Q* format arising from Pluronic L62 as a function of temperature, background corrected. All samples were prepared in D_2_O.

Figure 4 shows the scattering behaviour from foams stabilised by 0.05 wt % L62 (below its 25 °C CMC) at different temperature ranges (below and above the CMT ≈ 28 °C). Below the CMT (and CMC), a similar SANS pattern to the P123 is observed. The data fitting to the multilayer model has revealed a surfactant layer thickness (*L*) of 90 Å, spacing between layers (*D*) of 195 Å and that 6 layers (*M*) of the polymer are stacked at the interface. The change in the fit parameters are consistent with the change in the molecular weight and EO:PO ratio as we move from the bulkier P123 to the smaller L62.

As the temperature approaches the CMT, 25 °C, a broadening of the peak at mid *Q* ≈ 0.035 Å^−1^ can be observed. The temperature increase, namely between 37 °C and 45 °C, smears the mid *Q* peak rendering the fine features related to the air-water layer structures hard to define. However, these broad peaks indicate that micellar structures have been induced in the system and are present in the aqueous solution comprising the foam cell walls and further, that they dominate the scattering intensity. As an aside, the temperature induced micellisation also had a significant effect on the L62 foamability, as the temperature increased, the maximum foam height reached increased, Figure S8. 

## 4. Conclusions

It is our tenet that the (dynamic) interface creates novel structures in these non-ionic polymer/anionic surfactant systems, related to- but different from- those observed in the bulk phase, and similar to those observed in reflectivity studies on oppositely charged polymer/surfactant systems. The differentiating feature in these systems is the rate at which the various structures comes to equilibrium, contrasting with the age of the interface when characterized. Furthermore, it is clear that the air-water interface is more complex than originally thought, both under highly dynamic and equilibrium conditions [4,35,36,37]. Together, these studies demonstrate the importance of deriving parallel understanding of the complex interactions in the bulk and at interfaces, interfacial age, and how they drive the characteristics of the structures formed in the connected foam and solution phases.

## Figures and Tables

**Table 1:** Fit (multilayer model) parameters to the scattering data from foams stabilised by Pluronic P123, small molecule surfactants and their mixtures at concentrations below their CMC or mixed CMC. *L*: surfactant and/or polymer layer thickness, *M*: number of layers and *D*: spacing between the surfactant layers.

System Description	*L* (Å) ± 2	*M*	*D* (Å) ± 5
0.025 wt % P123 (*D11*)	140	4	180
0.025 wt % P123 (*SANS2d*)	140	5	390
7 mM C_12_TAB	40	5	180
0.025 wt % P123 + 0.1 mM C_12_TAB	140	4	180
4 mM SDS	35	5	180
0.025 wt % P123 + 0.1 mM SDS	120	5	410

## Supplementary Materials

The following are available online at http://www.mdpi.com/2073-4360/11/1/109/s1, Figure S1: Foam column apparatus used to study the foaming properties of polymer and surfactants solutions, Figure S2: SANS sample environment for studying foams. The foam was generated by pushing nitrogen gas through a 20 µm frit (A) at the base of a Perspex column (height of 25 cm, diameter of 4.6 cm), which contains approximately 50 mL of the surfactant solution. A 2 cm wide groove has been removed and covered with aluminium foil to allow the neutrons to cross the sample. The neutron beam impinges on the aluminium foil between (B) and (C) behind which the Perspex has been partially removed. For stable foams, the reservoir (D) collects the foam sample and returns it to the base via the plastic tube at (E). The cell was also be equipped with a controlled heating set up (heating jacket) at (F) and (G), Figure S3: Surface tension as a function of SDS and C_12_TAB concentration in water. All measurements were done at 25 °C, Figure S4: Surface tension as a function of P123 concentration water. All measurements were done at 25 °C, Figure S5: Surface tension as a function of SDS concentration in 0.025 wt % P123 and water. All measurements were done at 25 °C, Figure S6: Surface tension as a function C_12_TAB in 0.025 wt % P123 and water. All measurements were done at 25 °C, Figure S7: Surface tension from 2 wt % Pluronic L62 as a function of temperature in water, Figure S8: Maximum foam height of 2 wt % Pluronic L62 as a function of temperature. Dashed line is a guide to the eye, Figure S9: Small-angle scattering recast into an *I*(*Q*)**Q^n^* vs. *Q* format arising from foam stabilised by 0.025 wt % Pluronic P123 (circles), 0.025 wt % P123 + 0.1 mM h-SDS (hexagons) and 0.025 wt % P123 + 0.1 mM d-C_12_TAB (triangles). Data have been offset for clarity. The inset figure presents the raw data. Both the main figure and the inset are presented in a double logarithmic representation. All samples were prepared in D_2_O, Figure S10: Small-angle scattering recast into an *I*(*Q*)**Q^n^* vs. *Q* format arising from foam stabilised by 0.025 wt % Pluronic P123 (circles), 0.025 wt % P123 + 0.1 mM h-C_12_TAB (diamonds) and 0.025 wt % P123 + 0.1 mM d-C_12_TAB (hexagons). Data have been offset for clarity. The inset figure presents the raw data. Both the main figure and the inset are presented in a double logarithmic representation. All samples were prepared in D_2_O, Table S1: CMC and mixed CMC values from the small molecule surfactants, Pluronic P123 and their mixtures, Table S2: *d-spacing* (SANS) values from foams stabilised by Pluronic and small molecule surfactant mixtures.
