# Supplementary material for: Segregation versus Interdigitation in Highly Dynamic Polymer/Surfactant Layers
Source: Polymers (Basel). 2019 Jan 10;11(1):109. doi: 10.3390/polym11010109 (PMC6402036; doi:10.3390/polym11010109)
Supplement: Supplementary file 1 [file polymers-11-00109-s001.pdf]

polymers

Supplemental Materials: Segregation *versus* Interdigitation in Highly Dynamic Polymer/Surfactant Layers

Omar T. Mansour, Beatrice Cattoz, Manon Beaube, Richard K. Heenan, Ralf Schweins, Jamie Hurcom and Peter C. Griffiths

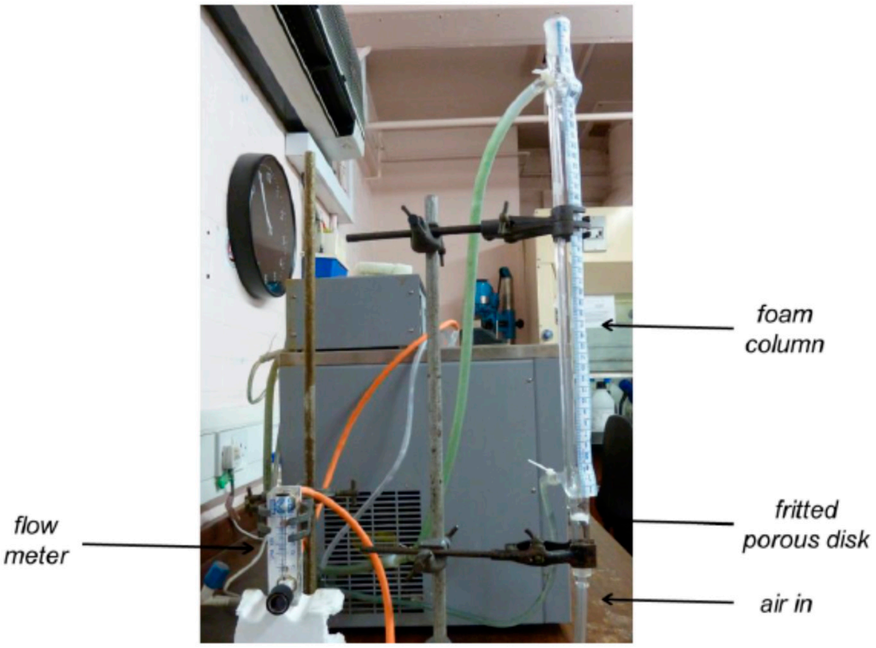

Figure S1. Foam column apparatus used to study the foaming properties of polymer and surfactants solutions.

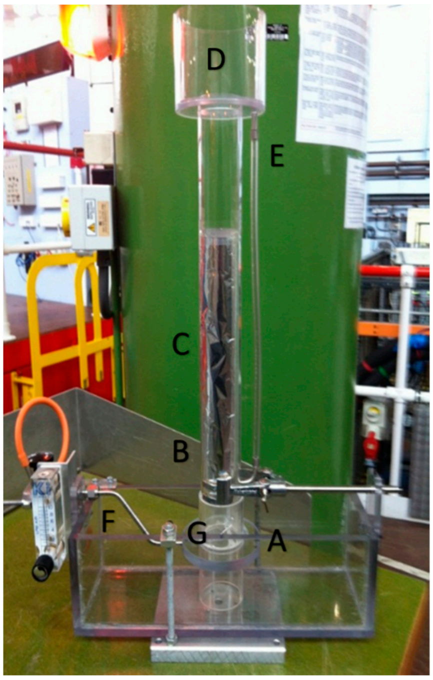

Figure S2. SANS sample environment for studying foams. The foam was generated by pushing nitrogen gas through a 20 µm frit (A) at the base of a Perspex column (height of 25 cm, diameter of 4.6 cm), which contains approximately 50 mL of the surfactant solution. A 2 cm wide groove has been removed and covered with aluminium foil to allow the neutrons to cross the sample. The neutron beam impinges on the aluminium foil between (B) and (C) behind which the Perspex has been partially removed. For stable foams, the reservoir (D) collects the foam sample and returns it to the base via the plastic tube at (E). The cell was also be equipped with a controlled heating set up (heating jacket) at (F) and (G).

Results

Surface tension measurements

The critical micelle concentrations (CMC) of the small molecule surfactants used in this work, SDS and $C_{12}TAB$, have been determined by recording the changes in the surface tension as a function of the surfactant concentration. The CMC is indicated by the break point in the data. The identified CMC for SDS was at ≈ 8 mM and for $C_{12}TAB$ was at ≈ 14 mM, both in agreement with literature values.

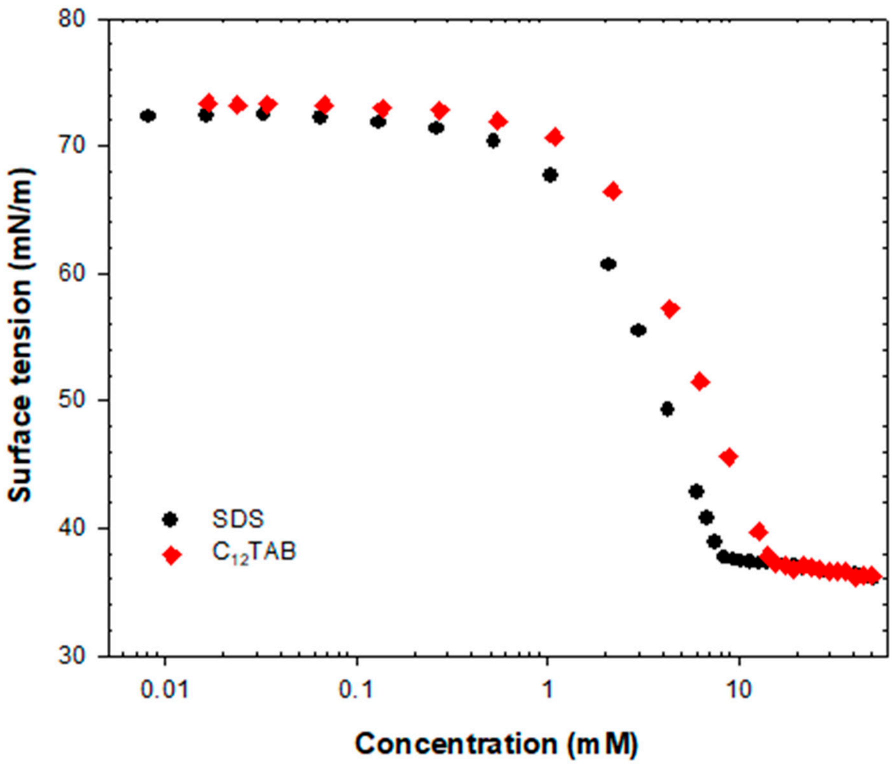

Figure S3. Surface tension as a function of SDS and $C_{12}TAB$ concentration in water. All measurements were done at 25 °C.

Similarly for the Pluronic, the CMC of P123 was also determined by surface tension, with the identified value of ≈ 0.05 wt%. The mixed CMC of P123 and SMS was also measured, and presented below. The CMT (≈28°C for Pluronic L62 was recorded by measuring the surface tension values as a function of temperature).

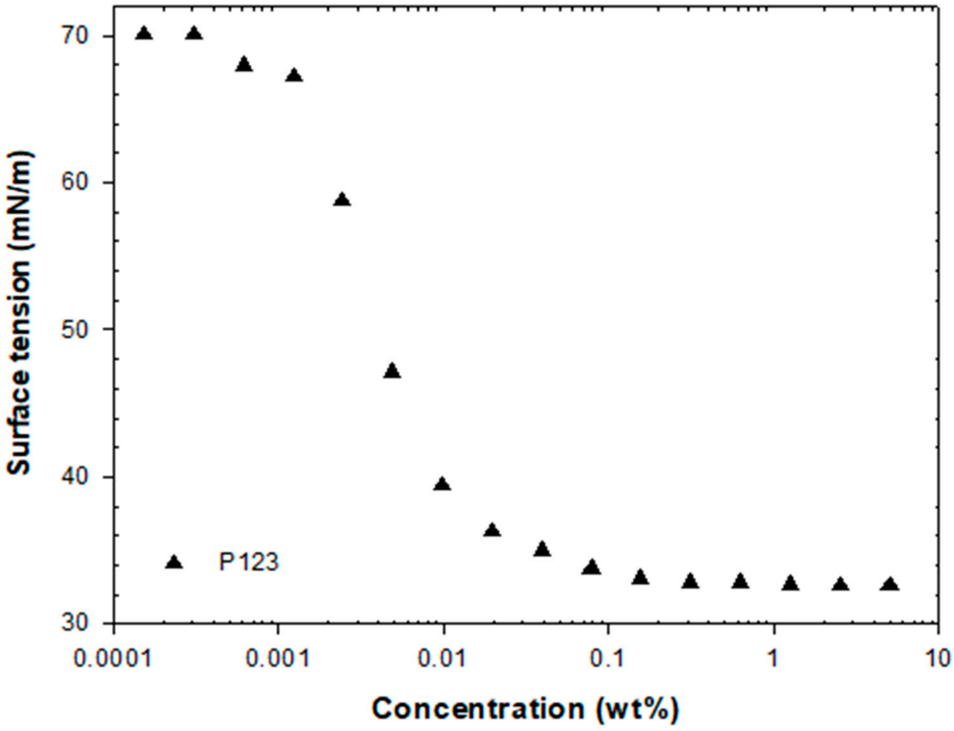

Figure S4. Surface tension as a function of P123 concentration water. All measurements were done at 25 °C.

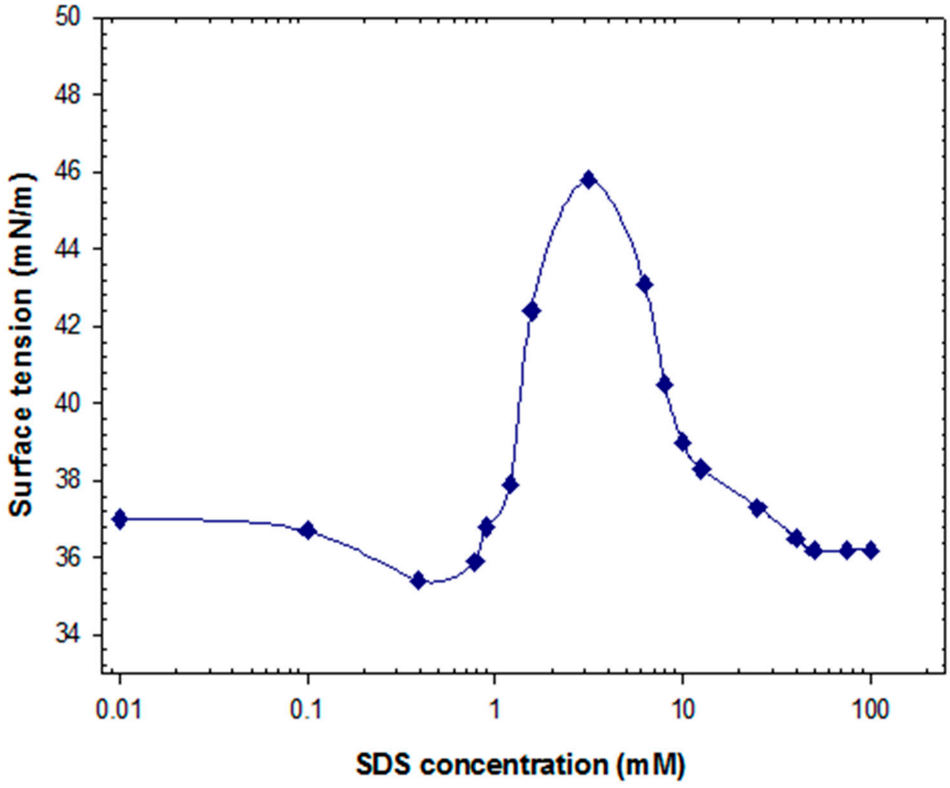

Figure S5. Surface tension as a function of SDS concentration in 0.025 wt % P123 and water. All measurements were done at 25 °C.

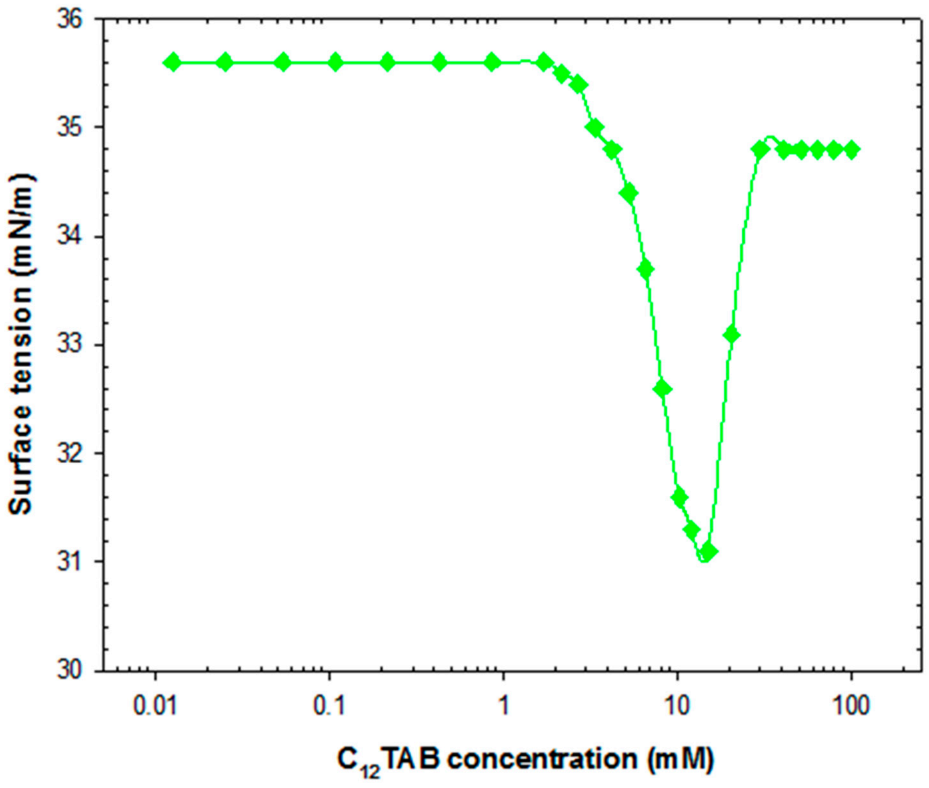

Figure S6. Surface tension as a function $C_{12}TAB$ in 0.025 wt % P123 and water. All measurements were done at 25 °C.

Table S1. CMC and mixed CMC values from the small molecule surfactants, Pluronic P123 and their mixtures.

System description	CMC	Mixed CMC
SDS in water	8.0 mM	-
$C_{12}TAB$ in water	14.0 mM	-
Pluronic P123 in water	0.05 wt%	-
0.025 wt % P123 + SDS in water	-	0.1 mM
0.025 wt % P123 + C12TAB in water	-	2.0 mM

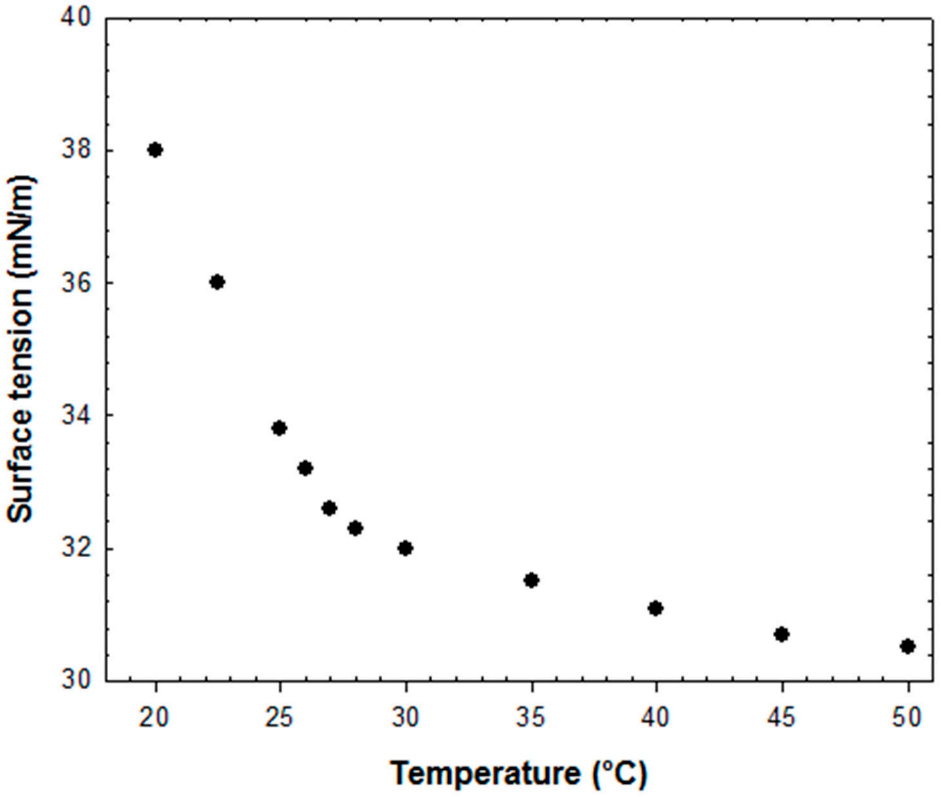

Figure S7. Surface tension from 2 wt % Pluronic L62 as a function of temperature in water.

Foam stability measurements

Foam stability measurement (foam half-life) as a function of Pluronic P123 and its mixture with the small molecule surfactants SDS and $C_{12}TAB$ are shown in Figure 2. For P123 at concentrations half of its measured CMC (0.025 wt%), we can notice that the decay in the stability profile is rapid with a half-life of ≈ 4250 seconds. Upon the addition of small concentration of SDS, 0.1 mM, the strong synergy between both components has significantly enhanced the foam stability, with a half-life that is now almost double of the P123 only (≈ 8000 seconds).

The weak interactions between P123 and $C_{12}TAB$ has also shown effect on the overall stability of the foam, where it has rendered the foam less stable when compared with the 0.025 wt% P123 foam case (half-life ≈ 3800 seconds). The change in the foaming behaviour observed here as the degree of interactions between various components was also observed by Petkova *et.al.*[1,2]

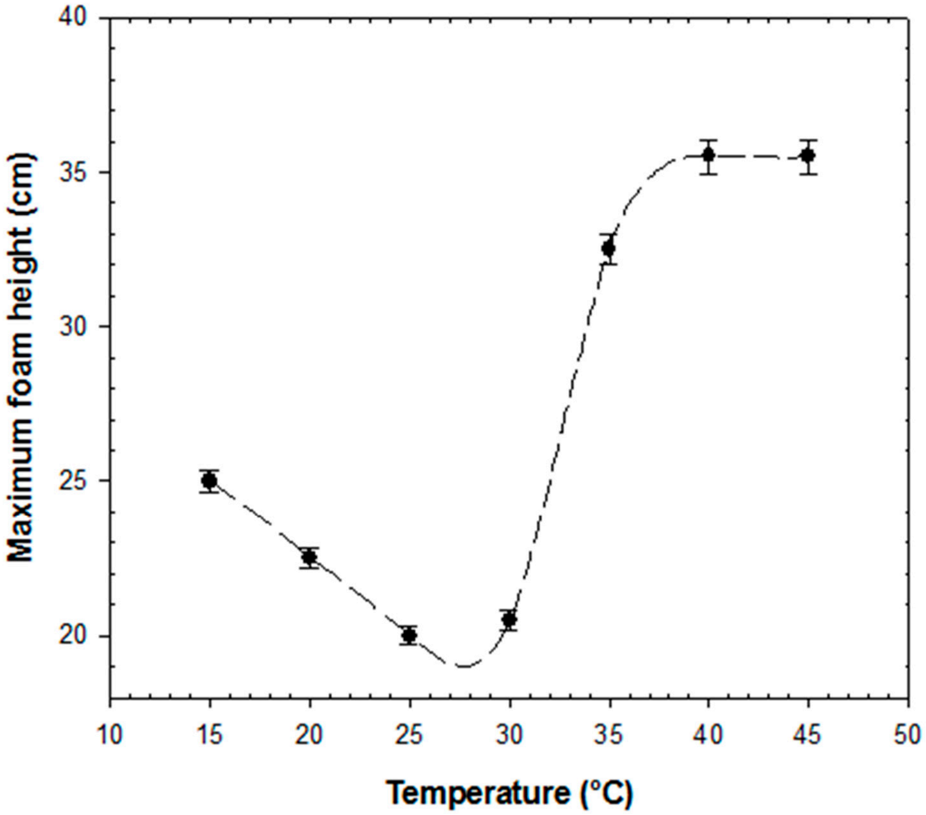

Figure S8. Maximum foam height of 2 wt % Pluronic L62 as a function of temperature. Dashed line is a guide to the eye.

Small-angle neutron scattering (SANS)

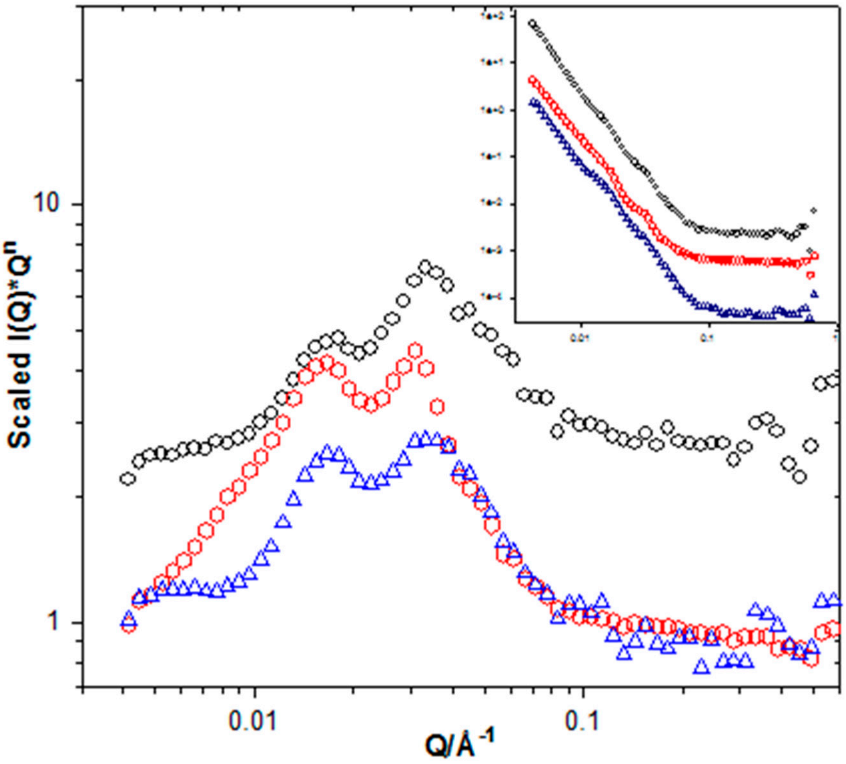

Figure S9. Small-angle scattering recast into an I(*Q*)**Q*ⁿ *vs. Q* format arising from foam stabilised by 0.025 wt % Pluronic P123 (circles), 0.025 wt % P123 + 0.1 mM h-SDS (hexagons) and 0.025 wt % P123 + 0.1 mM d-C_{12}TAB (triangles). Data have been offset for clarity. The inset figure presents the raw data. Both the main figure and the inset are presented in a double logarithmic representation. All samples were prepared in D_2O.

Table S2. d-spacing (SANS) values from foams stabilised by Pluronic and small molecule surfactant mixtures.

System description	d-spacing (Å)	
0.025 wt% P123	395	190
0.025 wt% P123 + h-SDS in D_2O	380	205
0.025 wt% P123 + d-SDS in D_2O	380	200
0.025 wt% P123 + h-C_{12}TAB in D_2O	380	195
0.025 wt% P123 + d-C_{12} h-TAB in D_2O	400	200

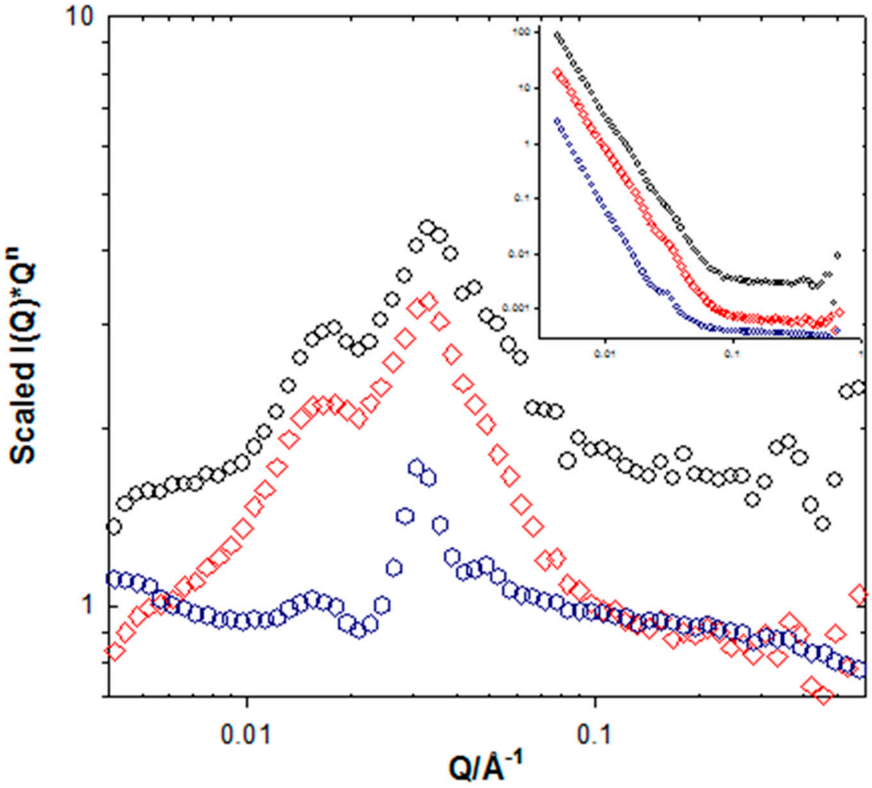

Figure S10. Small-angle scattering recast into an I(*Q*)**Q*^{n} *vs.* *Q* format arising from foam stabilised by 0.025 wt % Pluronic P123 (circles), 0.025 wt % P123 + 0.1 mM h-C_{12}TAB (diamonds) and 0.025 wt % P123 + 0.1 mM d-C_{12}TAB (hexagons). Data have been offset for clarity. The inset figure presents the raw data. Both the main figure and the inset are presented in a double logarithmic representation. All samples were prepared in D_2O.

References

1. R. Petkova, S. Tcholakova, N.D. Denkov, Foaming and foam stability for mixed polymer-surfactant solutions: Effects of surfactant type and polymer charge, *Langmuir* **2012,** 28, 4996–5009. doi:10.1021/la3003096.
2. R. Petkova, S. Tcholakova, N.D. Denkov, Role of polymer–surfactant interactions in foams: Effects of pH and surfactant head group for cationic polyvinylamine and anionic surfactants, *Colloids Surfaces A Physicochem. Eng. Asp.* **2013**, 1–12. doi:10.1016/j.colsurfa.2013.01.021.
